# *PTGER4* gene variant rs76523431 is a candidate risk factor for radiological joint damage in rheumatoid arthritis patients: a genetic study of six cohorts

**DOI:** 10.1186/s13075-015-0830-z

**Published:** 2015-11-05

**Authors:** Luis Rodriguez-Rodriguez, Jose Ivorra-Cortes, F. David Carmona, Javier Martín, Alejandro Balsa, Hanna W. van Steenbergen, Annette H. M. van der Helm-van Mil, Isidoro González-Álvaro, Benjamín Fernandez-Gutiérrez

**Affiliations:** Rheumatology Department and Heath Research Institute (IdISSC), Hospital Clinico San Carlos, c/o Prof. Martin Lagos s/n, 28040 Madrid, Spain; Rheumatology Department, Hospital Universitario y Politécnico La Fe, Valencia, Spain; Instituto de Parasitología y Biomedicina ‘López-Neyra’, CSIC, Granada, Spain; Rheumatology Department and Heath Research Institute (Idipaz), Hospital Universitario de La Paz, Madrid, Spain; Department of Rheumatology, Leiden University Medical Centre, Leiden, The Netherlands; Rheumatology Service and Heath Research Institute (IP), Hospital Universitario de La Princesa, Madrid, Spain

**Keywords:** Rheumatoid arthritis, Polymorphism, Radiological joint damage, *PTGER4*, rs76523431

## Abstract

**Introduction:**

Prostaglandin E receptor 4 (*PTGER4*) is implicated in immune regulation and bone metabolism. The aim of this study was to analyze its role in radiological joint damage in rheumatoid arthritis (RA).

**Methods:**

Six independent cohorts of patients with RA of European or North American descent were included, comprising 1789 patients with 5083 sets of X-rays. The Hospital Clínico San Carlos Rheumatoid Arthritis, Princesa Early Arthritis Register Longitudinal study, and Hospital Universitario de La Paz early arthritis (Spain) cohorts were used as discovery cohorts, and the Leiden Early Arthritis Clinic (The Netherlands), Wichita (United States), and National Databank for Rheumatic Diseases (United States and Canada) cohorts as replication cohorts. First, the *PTGER4* rs6896969 single-nucleotide polymorphism (SNP) was genotyped using TaqMan assays and available Illumina Immunochip data and studied in the discovery and replication cohorts. Second, the *PTGER4* gene and adjacent regions were analyzed using Immunochip genotyping data in the discovery cohorts. On the basis of pooled *p* values, linkage disequilibrium structure of the region, and location in regions with transcriptional properties, SNPs were selected for replication. The results from discovery, replication, and overall cohorts were pooled using inverse-variance–weighted meta-analysis. Influence of the polymorphisms on the overall radiological damage (constant effect) and on damage progression over time (time-varying effect) was analyzed.

**Results:**

The rs6896969 polymorphism showed a significant association with radiological damage in the constant effect pooled analysis of the discovery cohorts, although no significant association was observed in the replication cohorts or the overall pooled analysis. Regarding the analysis of the *PTGER4* region, 976 variants were analyzed in the discovery cohorts. From the constant and time-varying effect analyses, 12 and 20 SNPs, respectively, were selected for replication. Only the rs76523431 variant showed a significant association with radiographic progression in the time-varying effect pooled analysis of the discovery, replication, and overall cohorts. The overall pooled effect size was 1.10 (95 % confidence interval 1.05–1.14, *p* = 2.10 × 10^−5^), meaning that radiographic yearly progression was 10 % greater for each copy of the minor allele.

**Conclusions:**

The *PTGER4* gene is a candidate risk factor for radiological progression in RA.

**Electronic supplementary material:**

The online version of this article (doi:10.1186/s13075-015-0830-z) contains supplementary material, which is available to authorized users.

## Introduction

Rheumatoid arthritis (RA) is a systemic autoimmune disease that affects between 0.5 % and 1 % of the population in developed countries. It is a complex genetic condition with several patterns of progression [1] potentially associated with significant morbidity, disability, and costs to society [2]. Bearing this in mind, it would be useful to identify those patients at higher risk of severe disease, because early treatment could ameliorate its prognosis [3]. Genetic polymorphisms could be used as molecular biomarkers to predict disease development and to anticipate the clinical subset in which a particular patient will be included. Furthermore, radiological damage can be considered an objective measure of RA severity because the extent of joint destruction, measured using radiographic scores such as the Sharp/van der Heijde score (SHS), reflects the cumulative burden of inflammation.

Prostaglandin E receptor 4 (*PTGER4*, EP_4_), located at 5p13.1, encodes one of the four receptors identified for prostaglandin E_2_. This receptor is a member of the G protein–coupled receptor family, and it is expressed in several cell types, including T cells, macrophages, and synovial fibroblasts. This receptor has been implicated both in immune regulation and in bone metabolism. Together with another prostaglandin E_2_ receptor (EP_2_), it regulates the production of proinflammatory factors [such as interleukin (IL)-6, macrophage colony-stimulating factor, and vascular endothelial growth factor] in response to IL-1β in human synovial fibroblasts [4, 5]_._ This receptor also enhances T helper cell type 1 (Th1) differentiation and promotes Th17 cell expansion through the induction of IL-23 secretion by dendritic cells [6–8], leading to enhanced IL-17 expression and the accumulation of Th17 cells [6, 9]. Moreover, it was observed that the lack of EP_2_ and EP_4_ [5, 10] or the use of EP_4_ antagonists [7, 8] reduces the severity and suppresses the disease progression in mice subjected to experimentally induced arthritis and experimental autoimmune encephalomyelitis. EP_4_ also induces bone remodeling, both stimulating de novo bone formation [11] and, in parallel, increasing the number of osteoclasts through the induction of receptor activator of nuclear factor κB ligand [12, 13]. It also induces the production of parathyroid hormone-related peptide in RA fibroblasts treated with IL-1α [14].

Taking into account the pleiotropic effects of *PTGER4*, the objective of our present study was to assess the role of this receptor in RA disease severity. To that end, and considering the overlap of genetic loci between different immune-mediated diseases [15], we initially analyzed the association between the *PTGER4* rs6896969 single-nucleotide polymorphism (SNP) and radiological joint damage in patients with RA. This variant had previously been associated with multiple sclerosis [16]. Subsequently, we performed a more thorough analysis of the *PTGER4* region using available Immunochip data (Illumina, San Diego, CA, USA).

## Methods

### Study population

All the patients included in this study were of European or North American descent and had been diagnosed with RA according to the 1987 classification criteria of the American College of Rheumatology [17]. For the identification part of our study, we used three Spanish cohorts (referred to as the *discovery cohorts*): the Hospital Clínico San Carlos Rheumatoid Arthritis cohort (HCSC-RAC, Madrid, Spain) [18], the Princesa Early Arthritis Register Longitudinal study (PEARL, Madrid, Spain) [19], and the Hospital Universitario de La Paz early arthritis cohort (PAZ, Madrid, Spain) [20]. The discovery cohort comprised 525 patients with 1020 sets of X-rays. For the replication part of the study, we used three cohorts (referred to as the *replication cohorts*): the Leiden Early Arthritis Clinic cohort (EAC, Leiden, The Netherlands) [21], the Wichita cohort (Wichita, KS, USA) [22], and the National Databank for Rheumatic Diseases (NDB; United States and Canada) [23]. The replication cohorts comprised 1264 patients with 4063 sets of X-rays. Altogether, we included 1789 patients with 5083 sets of X-rays in this study.

Study approval was given by the local medical ethics committee of each participating center. Written informed consent was obtained from all participants.

### Variables

Radiographic joint damage was assessed using the SHSs of hands and wrists in the HCSC-RAC, PEARL, PAZ, Wichita, and NDB cohorts. SHSs of hands, wrist, and feet were obtained from the EAC cohort. This method scores both the presence and extent of erosions (scored in 16 areas of the hands and wrists on a scale of 0–5, and in 6 areas of the feet on a scale of 0–10), as well as narrowing and (sub)luxation of the joint (scored in 15 areas of the hands and wrists and in 6 areas of the feet, both on a scale of 0–4). The maximum erosion scores are 160 in hands and wrists and 120 in the feet, with maximum narrowing/(sub)luxation of 120 and 48, respectively. Total scores range from 0 to 280 for erosions and from 0 to 168 for narrowing/(sub)luxation. X-rays from each cohort were scored by different well-trained readers. Observers did not have access to the identity, clinic, or genetic data of the patients. The HCSC-RAC, PEARL, PAZ, EAC, and Wichita cohorts had serial X-rays that were chronologically scored, whereas the NDB cohort had only one set of X-rays per patient. The intraclass correlation coefficient (ICC) was assessed by twice reading 10 % of the radiographs. The ICCs of the HCSC-RAC, PEARL, PAZ, EAC, Wichita, and NDB cohorts were 0.99, 0.99, 0.98, 0.91, 0.98, and 0.98, respectively.

Patients’ clinical records were reviewed, and the following demographic and clinical variables were collected: sex, age at RA diagnosis, presence of rheumatoid factor (except for the Wichita and NDB cohorts), and anticitrullinated peptide antibodies (ACPA). Year at RA diagnosis was used as a surrogate marker for initial RA treatment in the HCSC-RAC, PEARL, PAZ, and EAC cohorts, as different strategies were implemented during different time periods. In the HCSC-RAC, PEARL, and PAZ cohorts, patients included before 1990 were initially treated with nonsteroidal anti-inflammatory drugs (NSAIDs). Between 1990 and 1999, initial treatment was monotherapy. Between 2000 and 2004, both combination therapy and biologic drugs started to be used. Beginning in 2005, the use of combination therapy and biologic drugs became widespread. In the EAC cohort, patients included in 1993–1995 were initially treated with NSAIDs; patients included in 1996–1998 were initially treated with hydroxychloroquine or sulfasalazine; and patients included in 1999–2006 were promptly treated with methotrexate [21]. Therefore, year at RA diagnosis was categorized for the HCSC-RAC, PEARL, and PAZ cohorts in the following periods: before 1990, 1990–1999, 2000-2004, and after 2004. For the EAC cohort, year at RA diagnosis was categorized as 1993–1995, 1996–1998, and 1999–2006. In the Wichita and NDB cohorts, year at RA diagnosis was not used as a surrogate marker for initial RA treatment, as most patients were diagnosed before the use of tailored therapy or biologics became widespread and initial treatment was rather homogeneous in each cohort.

### Genotyping

In the HCSC-RAC cohort, subjects were genotyped for the rs6896969 SNP using TaqMan Assays-on-Demand from Applied Biosystems (Foster City, CA, USA) according to the manufacturer’s protocol. The results were analyzed using the 7900HT Fast Real-Time PCR System (Applied Biosystems). Doubtful calls were manually checked.

Illumina Immunochip, a high-density throughput array designed to fine-map immune-related loci [24], was used to obtain genotyping data from the rs6896969 SNP in the PEARL, PAZ, EAC, Wichita, and NDB cohorts. Also, it was used to obtain genotyping data from the *PTGER4* gene and adjacent regions in all six cohorts. We selected a wide area around *PTGER4*, from 38,957,820 bp to 41,336,050 bp (GRCh37/hg19) (see Additional file 1: Figure S1). In the discovery cohorts, genotype data was quality-filtered using the following criteria: success call rate per individual and success call rate per SNP >0.95, minor allele frequency (MAF) >0.01, and Hardy–Weinberg equilibrium *p* value >0.001. In the replication cohorts, a similar approach was undertaken, except that samples with call rates less than 99.5 % and genotyping success rates less than 99 % were excluded [25].

To identify the potentially regulatory variants, we used publicly available databases and datasets, including RegulomeDB [26] and a dataset of expression quantitative trait loci studied in peripheral blood [27].

### Statistical analysis

Continuous variables were described using mean and standard deviation. Dichotomous and categorical variables were described using proportions. In all genetic analyses, an additive model of effect was used. SHS was log-transformed [log(SHS + 1)] to approximate a normal distribution.

The effect of the SNPs on radiological joint damage was assessed using two different models [21]. (1) In approach 1, we analyzed the overall effect of each polymorphism in radiological damage, assuming a stable effect over time (constant effect). In approach 2, we analyzed how the SNPs influenced the progression or the slope of the radiological joint damage over time (time-varying effect). Having this in mind, for datasets with multiple sets of X-rays per patient (HCSC-RAC, PEARL, PAZ, EAC, and Wichita) and to account for the within-patient correlation between measurements, linear mixed regression models were used [28, 29]. Approach 1 was carried out by analyzing the log-transformed SHS as the dependent variable and including the following in the model as independent variables: sex, age at symptom onset (in the discovery cohorts) or age at diagnosis (in the replication cohorts), initial RA treatment strategy (using the year at RA diagnosis as a surrogate marker; treat), elapsed time from RA symptom onset/diagnosis to the time of the X-ray (time), and SNP:

$$ SHS = \alpha + {\beta}_1 \times {\mathrm{sex}}_{\mathrm{j}} + {\beta}_2 \times {\mathrm{age}}_{\mathrm{j}} + {\beta}_3 \times {\mathrm{treat}}_{\mathrm{j}}+{\beta}_4 \times {\mathrm{time}}_{\mathrm{ij}} + {\beta}_5 \times {\mathrm{SNP}}_{\mathrm{j}} + {u}_{0\mathrm{j}} + {\varepsilon}_{\mathrm{ij}}, $$

where *i* represents X-ray; *j* represents patient; *ε*_*ij*_ is the normally distributed error with mean 0; *α*, *β*_1_, *β*_2_, *β*_3_, *β*_4,_ and *β*_5_ represent the intercept and the fixed effects coefficients for sex, age at symptom onset/diagnosis, initial treatment strategy, elapsed time from symptoms onset/diagnosis to X-ray, and polymorphism, respectively; and *u*_0j_ is the random effect per patient.

Approach 2 was carried out by introducing an interaction between elapsed time and polymorphism in approach 1:

$$ SHS = \alpha + {\beta}_1 \times {\mathrm{sex}}_{\mathrm{j}} + {\beta}_2 \times {\mathrm{age}}_{\mathrm{j}} + {\beta}_3 \times {\mathrm{treat}}_{\mathrm{j}} + {\beta}_4 \times {\mathrm{time}}_{\mathrm{ij}} + {\beta}_5 \times {\mathrm{SNP}}_{\mathrm{j}} + {\beta}_6 \times {\mathrm{time}}_{\mathrm{ij}} \times {\mathrm{SNP}}_{\mathrm{j}} + {u}_{0\mathrm{j}} + {\varepsilon}_{\mathrm{ij}}, $$

where *β*_6_ represents the coefficient for the interaction between elapsed time and polymorphism.

For the dataset with only one observation per patient (NDB cohort), approach 1 was carried out by analyzing the log-transformed SHS using linear regression models [30], adjusted by sex, age at diagnosis, elapsed time from RA diagnosis to the time of the X-ray, and SNP:

$$ SHS = \alpha + {\beta}_1 \times {\mathrm{sex}}_{\mathrm{j}} + {\beta}_2 \times {\mathrm{age}}_{\mathrm{j}} + {\beta}_3 \times {\mathrm{time}}_{\mathrm{ij}} + {\beta}_4 \times {\mathrm{SNP}}_{\mathrm{j}} + {\varepsilon}_{\mathrm{ij}} $$

Approach 2 was carried out using linear regression models with the estimated yearly progression rate (total SHS divided by number of disease year at the time of the X-ray; *YPR*) as the dependent variable and sex, age at diagnosis, and SNP as independent variables:

$$ YPR = \alpha + {\beta}_1 \times {\mathrm{sex}}_{\mathrm{j}} + {\beta}_2 \times {\mathrm{age}}_{\mathrm{j}} + {\beta}_3 \times {\mathrm{SNP}}_{\mathrm{j}} + {\varepsilon}_{\mathrm{ij}} $$

The results were back-transformed after analysis and expressed as effect size (ES) with 95 % confidence intervals (CIs). ES represents, in the constant effect analysis, a fold increase or decrease in radiological joint damage per copy of the minor allele that is constant over time, regardless of sex, age, elapsed time from inception to X-ray, or initial treatment (in HCSC-RAC, PEARL, PAZ, and EAC cohorts). In the time-varying effect analysis, the ES indicates the fold rate of radiological joint damage per year per copy of the minor allele compared with the reference common genotype, regardless of sex, age, elapsed time from inception to X-ray, or initial treatment.

The results from the discovery cohorts, the replication cohorts, and the six overall cohorts were pooled using inverse-variance–weighted meta-analysis to account for differences between cohorts [31]. Between-population heterogeneity was assessed by using the Durbin test and calculating the *I*^2^ statistic (percentage of total variation across studies that is due to heterogeneity rather than to chance). Fixed or random effects models were used according to the absence or presence of heterogeneity, respectively. A cutoff value ≥0.4 in the *I*^2^ statistic was used to define heterogeneity. The significance of the pooled β-coefficients was determined by using the *Z*-test, and 95 % CIs were calculated.

With regard to the pooled analysis of the rs6896969 polymorphism, the *p* value of the meta-analysis of the six overall cohorts was adjusted using the Bonferroni correction.

A fine-mapping analysis of the *PTGER4* region was performed in the HCSC-RAC, PEARL, and PAZ cohorts. We selected those variants to be replicated in the EAC, Wichita, and NDB cohorts, based on the *p* values of the pooled analysis of the discovery cohorts, on the linkage disequilibrium (LD) structure of the mapped region (see Additional file 1: Figures S2 and S3), and on the location of the variants in regions with known or predicted transcriptional regulatory properties: among those with a pooled *p* value <0.05, the one SNP for each LD block (*r*^2^ > 0.9) with the lowest RegulomeDB score (the lower the score, the higher the likelihood that the variant is ocated in a regulatory region).

To generate a *p*-value threshold for the pooled analysis of the 6 cohorts to retain an experiment-wide type I error of 0.05, we decided to carry out a Bonferroni correction based on the number of effectively independent SNPs tested, and in the fact that we performed a constant and a time-varying effect analysis for each SNP. LD blocks were defined by an *r*^2^ value >0.9 and a distance limit between SNPs of 500 kb. We observed 192 LD blocks, and because all polymorphisms were tested in two different models, the final threshold *p* value was 1.3 × 10^−4^. All analyses were performed using STATA version 12 (StataCorp, College Station, TX, USA) and IBM SPSS version 15.0 software (IBM, Armonk, NY, USA).

## Results

### Association between *PTGER4* rs6896969 SNP and radiographic joint destruction

**Fig. 1:**
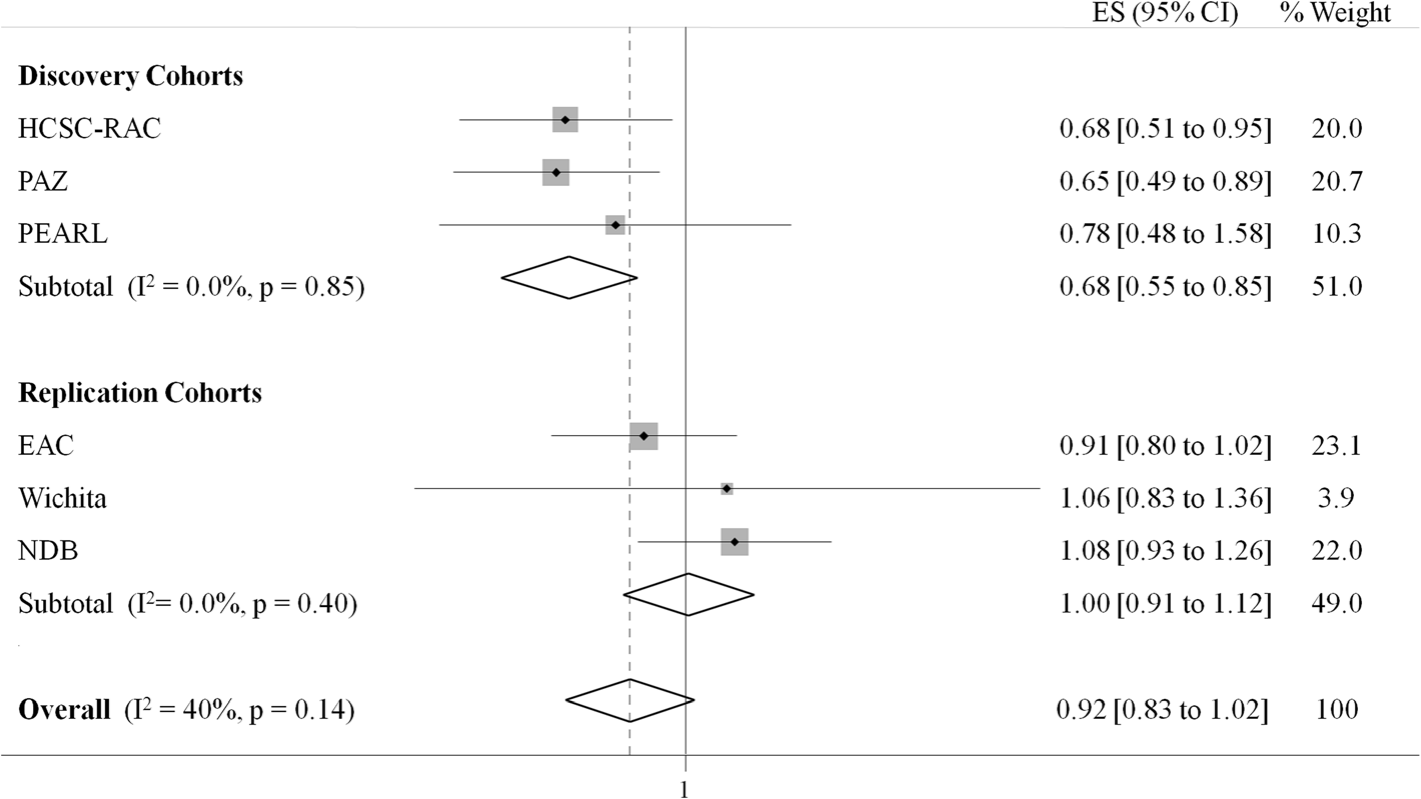
Forest plot representing the individual and pooled results of the effect of the *PTGER4* rs6896969 variant in radiographic joint destruction. *CI* confidence interval, *EAC* Leiden Early Arthritis Clinic cohort, *ES* effect size, *HCSC-RAC* Hospital Clínico San Carlos rheumatoid arthritis cohort, *NDB* National Databank for Rheumatic Diseases, *PAZ* Hospital Universitario de La Paz early arthritis cohort, *PEARL* Princesa Early Arthritis Register Longitudinal study, *PTGER4* prostaglandin E receptor 4

The demographic and clinical characteristics of the patients included in the study are shown in Table 1. We initially analyzed the effect of the *PTGER4* rs6896969 SNP on radiological joint damage in the HCSC-RAC, PEARL, and PAZ cohorts (Fig. 1). We observed that its minor allele was significantly associated with lower radiographic joint damage in the HCSC-RAC and PAZ cohorts (*p* = 0.03 and *p* = 0.02, respectively) in the constant effect analysis. The PEARL cohort also showed a protective effect, although it was not significant (*p* = 0.44). When we pooled the effects from the discovery cohorts, meta-analysis showed a significant protective association (fixed effects pooled *p* value = 8.6 × 10^−4^, *I*^2^ = 0). With regard to the time-varying effect analysis, no significant association was observed in any of the three cohorts (see Additional file 2: Table S1).

**Table 1:** Demographic and clinical characteristics of the patients with rheumatoid arthritis

	Cohort					
Variable	HCSC-RAC	PEARL	PAZ	EAC	Wichita	NDB
Total patients, *n*	402	81	42	597	100	567
Total sets of X-rays, *n*	637	210	173	3143	353	567
Female sex, *n* (%)	304 (75.62)	62 (76.54)	26 (61.90)	402 (67.3)	70 (70.0)	444 (78.3)
Age at diagnosis, yr, mean (SD)	57.09 (13.61)	55.39 (17.33)	53.11 (14.77)	57.1 (15.6)	49.1 (11.6)	48.7 (12.7)
Year at RA diagnosis, range	1976–2011	2000–2011	1992–2004	1993–2006	1963–1999	1980–1999
RF positivity, *n* (%)	260 (64.68)	47 (58.02)	35 (83.33)	342 (57.6)^a^	–	–
ACPA positivity, *n* (%)	167 (47.18)^b^	38 (47.50)^c^	33 (78.57)	309 (52.8)^d^	96 (97.0)^e^	452 (79.7)
Radiographic follow-up, yr	15	5	10	7	15	NA

*ACPA* anticitrullinated peptide antibodies, *EAC* Leiden Early Arthritis Clinic cohort, *HCSC-RAC* Hospital Clínico San Carlos rheumatoid arthritis cohort, *NDB* National Databank for Rheumatic Diseases cohort, *PAZ* Hospital Universitario de La Paz early arthritis cohort, *PEARL* Princesa Early Arthritis Register Longitudinal study cohort, *RA* rheumatoid arthritis, *RF* rheumatoid factor, *SD* standard deviation

^a^RF status missing in three patients from the EAC cohort

^b^ACPA status missing in 60 patients from the HCSC-RAC cohort

^c^ACPA status missing in one patient from the PEARL cohort

^d^ACPA status missing in 12 patients from the EAC cohort

^e^ACPA status missing in one patients from the Wichita cohort

We attempted to validate the results from the constant effect analysis in our replication cohorts (EAC, Wichita, and NDB) (Fig. 1). None of them showed a significant association between the rs6896969 variant and radiological joint damage (*p* = 0.10, *p* = 0.63, and *p* = 0.32, respectively). Moreover, only in the EAC cohort did the polymorphism show a protective effect. When we pooled the results from the replication cohorts, no significant association was observed (fixed effects model *p* value = 0.92, *I*^2^ = 0). Furthermore, when we combined the six cohorts, no significant association was observed (random effects model *p* value = 0.10, *I*^2^ = 0.46) (Fig. 1).

### Analysis of the *PTGER4* region and radiological joint damage

Because rs6896969 showed a significant association in the pooled analysis of the Spanish cohorts, and because we considered *PTGER4* a good candidate gene to play a part in RA radiological joint damage due to its immune regulation and bone metabolism roles, we decided to carry on and perform a fine-mapping analysis of the region around *PTGER4* using available Immunochip data. Our aim was not to refine the signal from rs6896969, as this SNP did not show a significant association in the overall pooled analysis, but to detect other variant(s) that might be significantly associated with radiological joint damage. Moreover, we decided not to restrict our analysis to the constant effect models, although this was the model that showed a significant association with radiological joint damage in the Spanish cohorts.

A total of 976 SNPs were analyzed in the discovery cohorts (HCSC-RAC, PEARL, and PAZ). In the pooled analysis, we observed 77 variants associated with radiological joint damage in the constant effect analysis and 91 SNPs in the time-varying effect analysis, with a pooled *p* value <0.05 (see Additional file 2: Tables S2 and S3). Considering the LD pattern, 11 SNPs captured all 77 constant effect analysis variants (with a mean *r*^2^ of 0.98) (Additional file 2: Table S4). We decided to include rs1876143 as well because, although another polymorphism from its LD block (rs1876140) captured the variability better, the former had a much lower RegulomeDB score. In the pooled analysis of the replication cohorts or in the pooled analysis of all six cohorts, no variant showed a significant association with radiological joint damage (Table 2).

**Table 2:** Candidate single-nucleotide polymorphisms selected for replication from constant effect analysis

				Discovery cohort			Replication cohort			Overall cohort (*n* = 6)		
SNP	Position	RegulomeDB score	Westra et al. [27] *cis-eQTL p* value (gene)	Pooled ES (95 % CI)	Pooled *p* value^a^	*I* ^2^ value	Pooled ES (95 % CI)	Pooled *p* value^a^	*I* ^2^ value	Pooled ES (95 % CI)	Pooled *p*-value^a^	*I* ^2^ value
rs348561	40312392	5	–	0.82 (0.67–0.996)	0.05	0.03	0.99 (0.90–1.08)	0.81	0	0.94 (0.86–1.04)	0.23	0.23
rs115430320	40318274	–	–	0.55 (0.39–0.80)	0.001	0	0.96 (0.80–1.15)	0.68	0.34	0.86 (0.74–1.02)	0.19	0.51
rs348601	40320006	5	–	0.86 (0.74–0.999)	0.05	0	0.94 (0.87–1.02)	0.45	0.42	0.92 (0.86–0.99)	0.03	0.23
rs394213	40333751	3a	–	0.76 (0.63–0.91)	0.003	0	0.97 (0.88–1.06)	0.45	0	0.92 (0.85–1.0005)	0.05	0.35
rs35870239	40344952	–	–	0.74 (0.59–0.93)	0.01	0.15	0.98 (0.88–1.09)	0.69	0	0.93 (0.84–1.02)	0.13	0.35
rs62359777	40350759	6	–	0.81 (0.68–0.95)	0.01	0	0.96 (0.86–1.08)	0.50	–	0.91 (0.83–0.999)	0.05	0.38
rs75248677	40365721	5	–	0.71 (0.56–0.90)	0.005	0.38	0.94 (0.84–1.07)	0.35	0	0.89 (0.80–0.99)	0.04	0.36
rs56049341	40376614	5	–	2.44 (1.28–4.66)	0.01	0	1.34 (0.72–2.51)	0.36	–	1.79 (1.14–2.81)	0.01	0
rs56027413	40382134	2b	–	1.18 (1.003–1.40)	0.05	0	1.03 (0.94–1.12)	0.55	0.19	1.06 (0.98–1.15)	0.14	0.02
rs12520940	40382428	4	–	0.81 (0.67–0.98)	0.03	0.33	1.00 (0.91–1.10)	0.98	0	0.96 (0.88–1.05)	0.35	0.31
rs1876140	40505752	–	3.68x10^–15^ (*PTGER4*)	0.80 (0.66–0.97)	0.03	0	1.05 (0.96–1.16)	0.36	0.43	0.997 (0.91–1.09)	0.75	0.52
rs1876143	40521648	2b	7.32x10^–13^ (*PTGER4*)	0.80 (0.66–0.96)	0.02	0	1.06 (0.96–1.17)	0.25	0.12	0.999 (0.92–1.09)	0.68	0.52

*ES* effect size, *PTGER4* prostaglandin E receptor 4, *SNP* single-nucleotide polymorphism

Data are based on pooled analysis in the discovery cohort, replication cohort, and all six overall cohorts

^a^Random effects *p* value was used if *I* ^2^ value was ≥0.4. Otherwise, fixed effects *p* value was used

**Fig. 2:**
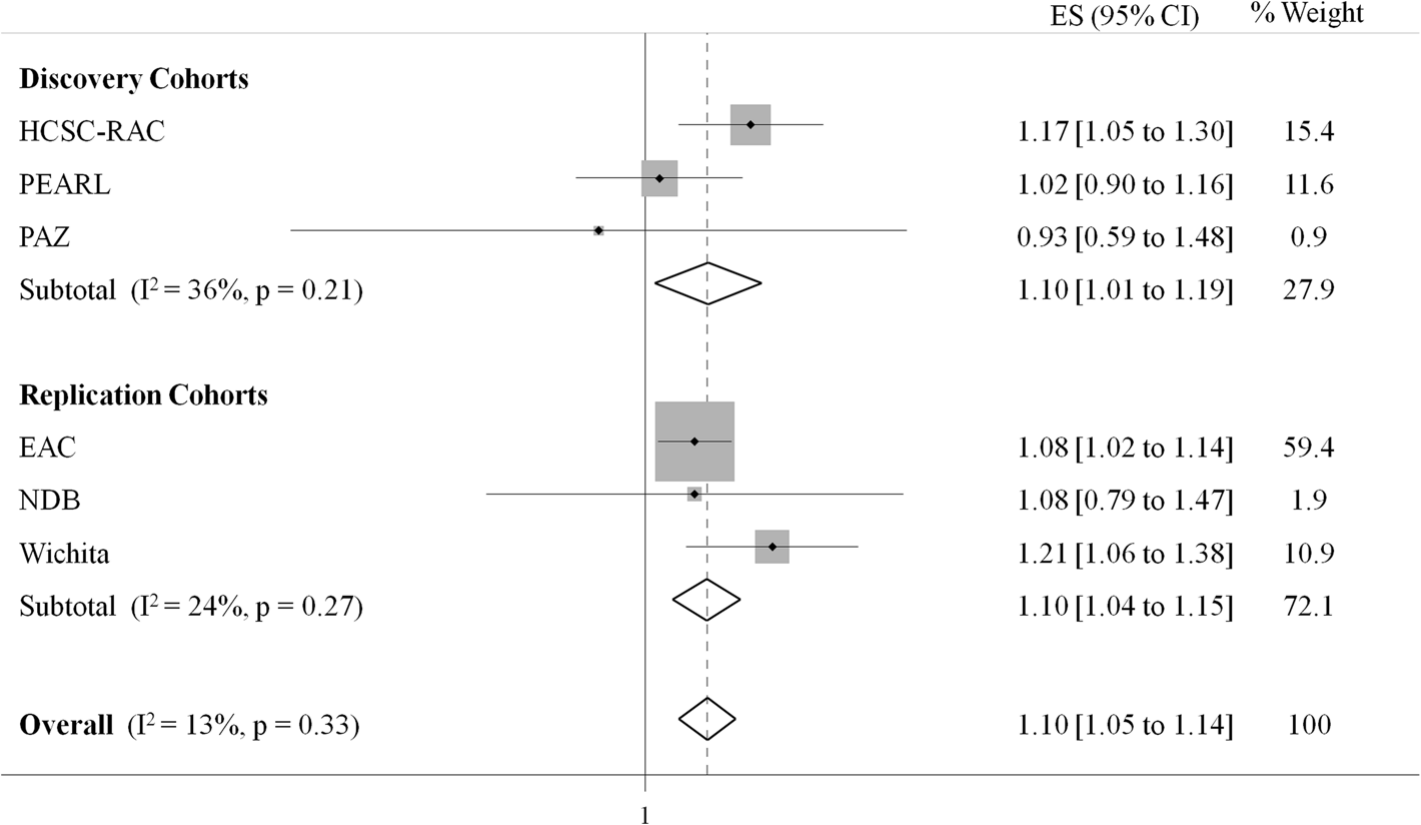
Forest plot representing the individual and pooled results of the effect of the *PTGER4* rs76523431 variant in radiographic joint destruction. *CI* confidence interval, *EAC* Leiden Early Arthritis Clinic cohort, *ES* effect size, *HCSC-RAC* Hospital Clínico San Carlos rheumatoid arthritis cohort, *NDB* National Databank for Rheumatic Diseases, *PAZ* Hospital Universitario de La Paz early arthritis cohort, *PEARL* Princesa Early Arthritis Register Longitudinal study, *PTGER4* prostaglandin E receptor 4

With regard to the time-varying effect analysis, 19 SNPs captured all 91 variants (with a mean *r*^2^ of 0.99) (Additional file 2: Table S5). We decided to include rs4409138 as well because it has a lower RegulomeDB score compared with the variant that captures the variability for the LD block. In the pooled analysis of the replication cohorts, three SNPs showed a *p* value <0.05 (Table 3). In the pooled analysis of the six overall cohorts, we observed that only the rs76523431 variant showed a significant association with radiological joint damage below the threshold *p* value (fixed effects model *p* value = 2.10 × 10^−5^, *I*^2^ = 0.13) (Fig. 2). We observed an ES of 1.10 (95 % CI 1.05–1.14), meaning that radiographic yearly progression was 10 % greater for each copy of the minor allele.

**Table 3:** Candidate single-nucleotide polymorphisms selected for replication from the time-varying effect analysis

				Discovery cohort			Replication cohort			Overall cohort (*n* = 6)		
SNP	Position	RegulomeDB score	Westra et al. [27] *cis p* value (gene)	Pooled ES (95 % CI)	Pooled *p* value^a^	*I* ^2^ value	Pooled ES (95 % CI)	Pooled *p* value^a^	*I* ^2^ value	Pooled ES (95 % CI)	Pooled *p* value^a^	*I* ^2^ value
rs80058440	40350930	6	–	1.05 (1.01–1.08)	0.01	0	0.99 (0.97–1.02)	0.62	–	1.01 (0.99–1.03)	0.18	0.50
rs6451489	40357663	5	–	0.97 (0.95–0.99)	0.01	0.15	1.01 (0.99–1.02)	0.40	0	0.99 (0.98–1.01)	0.46	0.43
rs12515934	40376930	2b	–	1.05 (1.01–1.08)	0.004	0.27	1.01 (0.98–1.03)	0.54	0	1.02 (1.002–1.04)	0.03	0.24
rs12520940	40382428	4	–	1.04 (1.01–1.07)	0.01	0	0.99 (0.97–1.01)	0.25	0	1.003 (0.99–1.02)	0.53	0.47
rs13160782	40428061	4	–	1.04 (1.01–1.06)	0.01	0	1.01 (1.00–1.03)	0.16	0.28	1.02 (1.005–1.03)	0.01	0.05
rs76523431	40443657	6	–	1.10 (1.01–1.19)	0.02	0.36	1.10 (1.04–1.15)	3.32 × 10^−4^	0.24	1.10 (1.05–1.14)	2.10 × 10^−5^	0.13
rs78607701	40447491	4	–	0.93 (0.89–0.98)	0.004	0	0.99 (0.95–1.03)	0.51	0	0.97 (0.94–0.99)	0.02	0
rs58752461	40481432	4	–	0.97 (0.94–0.99)	0.03	0	0.98 (0.96–1.00)	0.08	0	0.98 (0.96–0.99)	0.01	0
rs4587119	40492734	–	1.16 × 10^−16^ (*PTGER4*)	1.04 (1.004–1.07)	0.03	0.31	1.00 (0.98–1.03)	0.87	0	1.01 (0.99–1.03)	0.14	0.27
rs13181935	40493646	–	6.88 × 10^−11^ (*PTGER4*)	1.05 (1.01–1.08)	0.01	0	1.01 (0.98–1.03)	0.61	0	1.02 (1.0002–1.04)	0.05	0.17
rs7707931	40496930	6	3.68 × 10^–13^ (*PTGER4*)	1.03 (1.004–1.06)	0.02	0	1.01 (0.99–1.02)	0.58	0	1.01 (0.99–1.03)	0.08	0
rs74630635	40510535	–	–	1.05 (1.01–1.10)	0.02	0	1.05 (1.01–1.09)	0.01	0.22	1.05 (1.02–1.08)	6.40 × 10^−4^	0
rs7705019	40587804	3a	–	1.04 (1.01–1.08)	0.03	0.49	1.01 (0.99–1.03)	0.40	0	1.02 (1.003–1.04)	0.03	0.36
rs2174550	40602383	–	–	0.93 (0.88–0.99)	0.04	0.33	1.00 (0.98–1.03)	0.83	0.44	0.99 (0.97–1.02)	0.15	0.53
rs4409138	40603605	2b	–	1.04 (1.01–1.07)	0.01	0.37	1.01 (0.99–1.03)	0.37	0	1.02 (1.002–1.04)	0.03	0.24
rs924967	40615122	–	2.36 × 10^−11^ (*PTGER4*)	1.04 (1.01–1.07)	0.01	0.26	1.01 (0.99–1.03)	0.38	0	1.02 (1.002–1.04)	0.03	0.18
rs76504641	40624582	6	–	0.96 (0.92–0.99)	0.02	0	1.00 (0.98–1.02)	0.98	0	0.99 (0.97–1.01)	0.28	0.35
rs4432939	40671099	–	–	0.97 (0.95–0.99)	0.03	0	0.98 (0.96–1.00)	0.05	0	0.98 (0.96–0.99)	0.004	0
rs45480797	40680964	2a	–	0.95 (0.91–0.99)	0.01	0	0.98 (0.95–1.01)	0.24	0	0.97 (0.95–0.99)	0.02	0
rs78733746	40727730	4	–	0.93 (0.88–0.99)	0.02	0	1.00 (0.96–1.04)	0.93	0	0.98 (0.95–1.01)	0.19	0

*CI* confidence interval, *ES* effect size, *PTGER4* prostaglandin E receptor 4, *SNP* single-nucleotide polymorphism

Data are based on pooled analysis in the discovery cohort, replication cohort, and all six overall cohorts

^a^Random effects *p* value was used if *I* ^2^ value was ≥0.4. Otherwise, fixed effects *p*-value was used

## Discussion

We analyzed, for the first time to our knowledge, the potential implication of the *PTGER4* gene in RA disease severity, measured as radiological joint damage in several cohorts of Caucasian patients. In this study, we observed that the rs76523431 variant showed a positive and significant association with disease severity in the time-varying effect analysis: the more copies of the minor allele, the greater the radiographic progression (more joint damage per time unit).

Taking into account the role played by EP_4_ in the differentiation of Th1 lymphocytes and in the expansion of Th17 cells [7, 8] (both implicated in RA pathogenesis [32]), and that the lack of *Ptger4* or its blockage ameliorates the disease in several mouse arthritis models (collagen-induced [5, 8], collagen antibody–induced [10], and glucose-6-phosphate isomerase–induced arthritis models [8]), we decided to analyze the role of *PTGER4* in RA severity. Several SNPs from this gene or its surrounding region had previously been associated with other immune-regulated diseases. rs10440635 was associated with ankylosing spondylitis (AS) [9] in Caucasians and with AS severity [33] in Chinese patients. Many variants have been associated with inflammatory bowel disease. rs11742570 [34–36], rs17234657 [37–39], rs4495224 [40], rs7720838 [40], rs4613763 [41], rs9292777 [41, 42], and rs1373692 [43, 44] are risk factors for Crohn’s disease, although some associations have not always been replicated [45]. However, rs4613763 [46, 47] and rs6451493 [48] have been associated with ulcerative colitis. Several variants have also been associated with multiple sclerosis (rs9292777 [49] and rs4613763 [50]), including rs6896969 [16], the SNP initially analyzed in the present study. The influence of rs17234657 and rs6871834 in RA risk was also analyzed, with no significant results observed [38].

With regard to the variant significantly associated with radiographic progression in this study, it has not previously been associated with any other immune-mediated diseases. Moreover, other SNPs in complete LD with rs76523431 (rs114152040, rs113233093, rs116154382, and rs112110624) also have not previously been associated with any condition (data obtained from SNAP in 1000 Genomes Project Pilot 1 [51]). However, one of those SNPs (rs112110624) is located in the binding site of two transcription factors (TFAP2A and TFAP2C) [26]. It is possible that this variant affects the binding of any of these transcription factors, and therefore it could alter the expression of EP_4_. We could speculate that rs112110624 is the polymorphism driving the association of *PTGER4* with RA severity, although it cannot be ruled out that rs76523431 or any other SNP in LD is the real causal variant of this association. We also used miRBASE [52] to assess if any SNP was located in or near a described miRNA. Unfortunately, no miRNA has been described in that region.

With regard to the limitations of our study, it is important to point out the presence of clinical heterogeneity among cohorts, such as differences in cohort size, number of X-rays per patient, year of disease onset, presence of ACPA, follow-up duration, and whether the feet were included in the radiological joint damage assessment. To account for these factors, first we analyzed each cohort individually, adjusting for most of these variables. Then we combined the results with inverse-variance–weighted meta-analysis, which gives, in the pooled results, a higher weight to those cohorts with more precise results.

Also, X-rays in some cohorts were obtained at standardized time points (PEARL, PAZ, EAC, and Wichita), whereas in others (HCSC-RAC and NDB) X-rays were taken when requested by the patient’s rheumatologist as deemed necessary and not as part of a protocol.

Finally, the *PTGER4* rs76523431 variant has a low MAF (HCSC-RAC 1.8 %, PEARL 4.3 %, PAZ 1.2 %, EAC 2.1 %, NDB 2.0 %, and Wichita 1.5 %), and therefore it has a lower power to detect weak effects than loci with higher MAFs. However, it is important to take into account that this is an exploratory result, and future studies need to be performed to verify that this is not a spurious association.

## Conclusions

The results reported in this article suggest that the *PTGER4* variant rs76523431 could be associated with greater radiographic progression in Caucasian patients with RA. Further studies are necessary to fully elucidate the effect of this and other SNPs in *PTGER4* expression.

## Additional files

Additional file 1:
**Figure S1.** Region analyzed in the fine-mapping analysis. **Figure S2.** Linkage disequilibrium blocks among the significant SNPs (pooled *p* < 0.05) from the constant effect analysis. **Figure S3.** Linkage disequilibrium blocks among the significant SNPs (pooled *p* < 0.05) from the time-varying effect analysis. (DOC 845 kb) Additional file 2:
**Table S1.** Multivariate linear mixed models to analyze the time-varying effect of the *PTGER4* rs6896969 variant in radiographic joint damage of the HCSC-RAC, PEARL, and PAZ cohorts. **Table S2.** Single-nucleotide polymorphisms with *p* < 0.05 in the constant effect pooled analysis from the discovery cohorts. **Table S3.** Single-nucleotide polymorphisms with *p* < 0.05 in the time-varying effect pooled analysis from the discovery cohorts. **Table S4.** Single-nucleotide polymorphisms selected for replication in the constant effect analysis and their associations with radiological joint damage in the six cohorts. **Table S5. **Single-nucleotide polymorphisms selected for replication in the time-varying effect analysis and their associations with radiological joint damage in the six cohorts. (DOC 325 kb)

## Abbreviations

ACPA: anticitrullinated peptide antibodies
AS: ankylosing spondylitis
CI: confidence interval
EAC: Leiden Early Arthritis Clinic cohort
EP_2_: prostaglandin E_2_ receptor
EP_4_: prostaglandin E receptor 4
ES: effect size
HCSC-RAC: Hospital Clínico San Carlos rheumatoid arthritis cohort
ICC: intraclass correlation coefficient
IL: interleukin
LD: linkage disequilibrium
MAF: minor allele frequency
NDB: National Databank for Rheumatic Diseases
NSAID: nonsteroidal anti-inflammatory drugs
PAZ: Hospital Universitario de La Paz early arthritis cohort
PEARL: Princesa Early Arthritis Register Longitudinal study
*PTGER4*: prostaglandin E receptor 4
RA: rheumatoid arthritis
RF: rheumatoid factor
SD: standard deviation
SHS: Sharp/van der Heijde score
SNP: single-nucleotide polymorphism
Th: T helper cell
