# Supplementary material for: PTGER4 gene variant rs76523431 is a candidate risk factor for radiological joint damage in rheumatoid arthritis patients: a genetic study of six cohorts
Source: Arthritis Res Ther. 2015 Nov 5;17:306. doi: 10.1186/s13075-015-0830-z (PMC4634155; doi:10.1186/s13075-015-0830-z)

**Figure S1**: Region analyzed in the fine-mapping analysis.


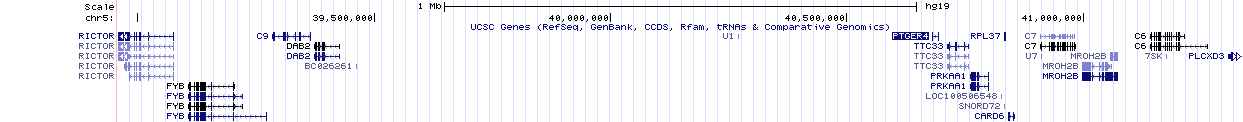


**Figure S2**: Linkage disequilibrium blocks among the significant SNPs (pooled p<0.05) from the *constant effect* analysis.


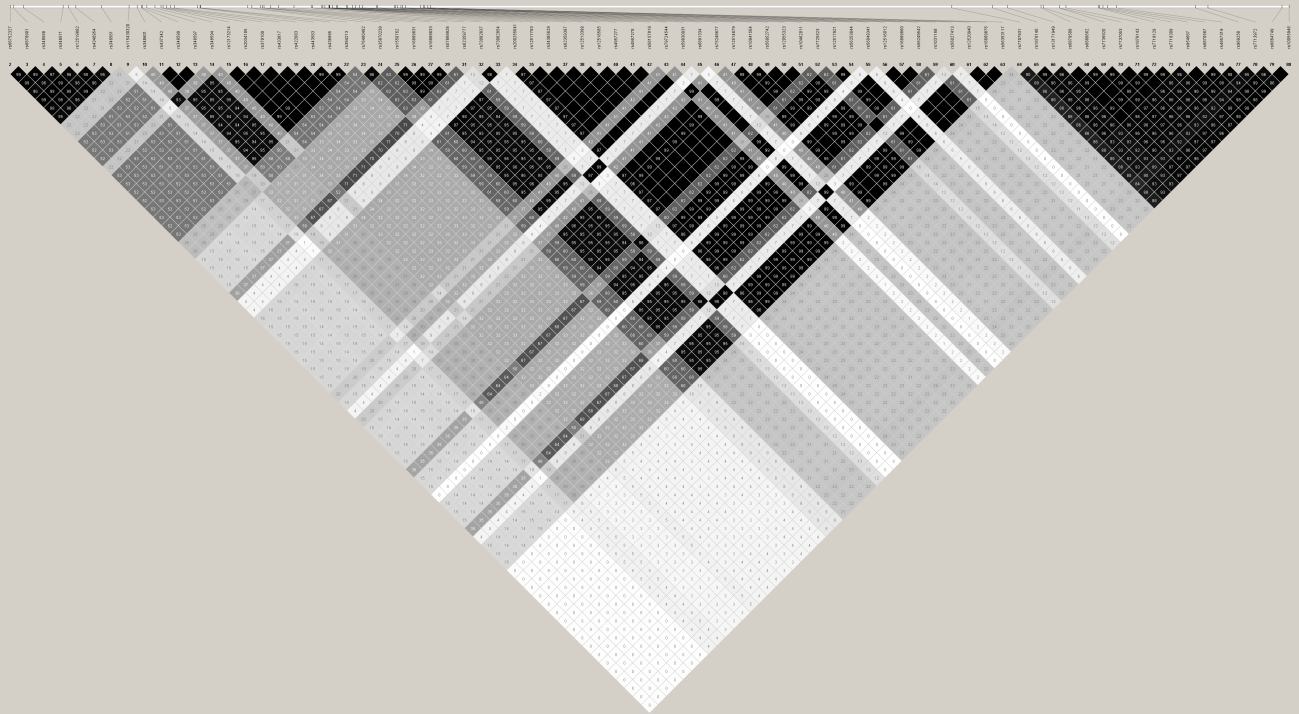


**Figure S3**: Linkage disequilibrium blocks among the significant SNPs (pooled p<0.05) from the *time-varying effect* analysis.


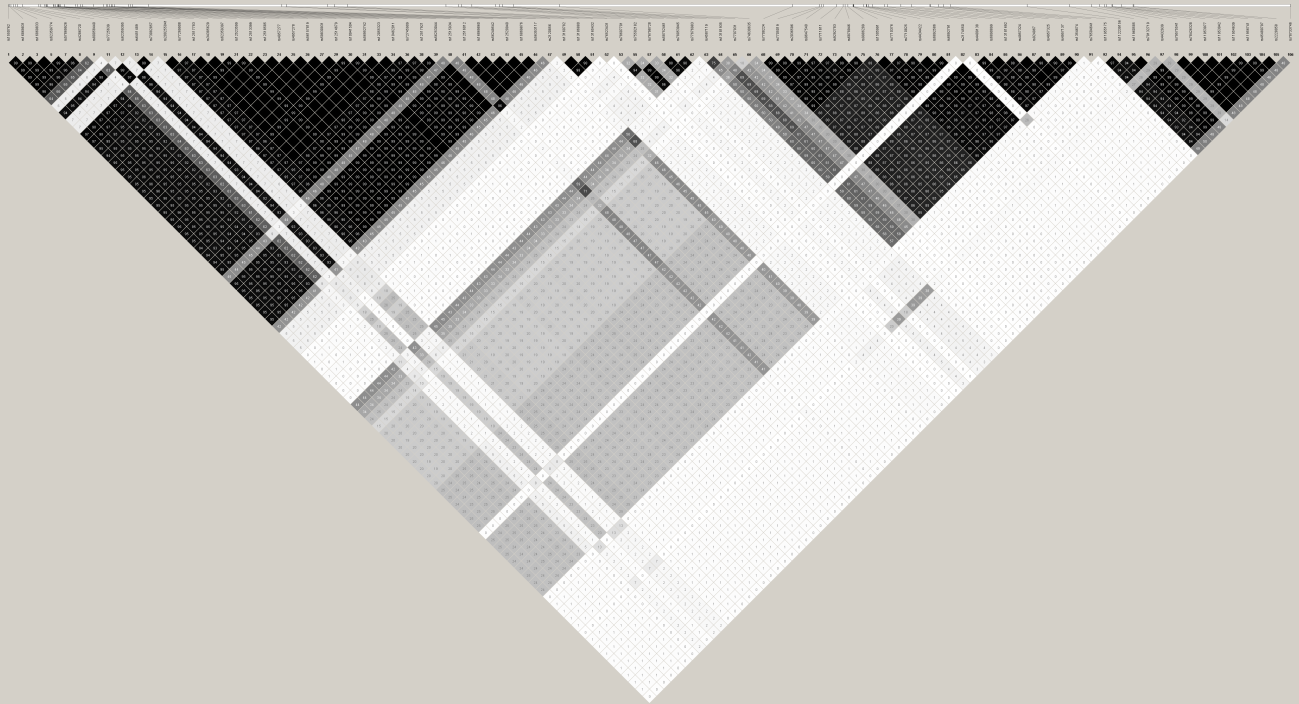

Supplement: Additional file 1: — Figure S1. Region analyzed in the fine-mapping analysis. Figure S2. Linkage disequilibrium blocks among the significant SNPs (pooled p < 0.05) from the constant effect analysis. Figure S3. Linkage disequilibrium blocks among the significant SNPs (pooled p < 0.05) from the time-varying effect analysis. (DOC 845 kb) [file 13075_2015_830_MOESM1_ESM.doc]
