# Supplementary material for: PTGER4 gene variant rs76523431 is a candidate risk factor for radiological joint damage in rheumatoid arthritis patients: a genetic study of six cohorts
Source: Arthritis Res Ther. 2015 Nov 5;17:306. doi: 10.1186/s13075-015-0830-z (PMC4634155; doi:10.1186/s13075-015-0830-z)
Supplement: Additional file 2: — Table S1. Multivariate linear mixed models to analyze the time-varying effect of the PTGER4 rs6896969 variant in radiographic joint damage of the HCSC-RAC, PEARL, and PAZ cohorts. Table S2. Single-nucleotide polymorphisms with p < 0.05 in the constant effect pooled analysis from the discovery cohorts. Table S3. Single-nucleotide polymorphisms with p < 0.05 in the time-varying effect pooled analysis from the discovery cohorts. Table S4. Single-nucleotide polymorphisms selected for replication in the constant effect analysis and their associations with radiological joint damage in the six cohorts. Table S5. Single-nucleotide polymorphisms selected for replication in the time-varying effect analysis and their associations with radiological joint damage in the six cohorts. (DOC 325 kb) [file 13075_2015_830_MOESM2_ESM.doc]

**Table S1**: Multivariate linear mixed models to analyze the *time-varying* *effect* of the *PTGER4* rs6896969 variant in radiographic joint damage of the HCSC-RAC, PEARL and PAZ.

| Cohort | ES [95%CI] | p |
| --- | --- | --- |
| HCSC-RAC | 1.02 [0.98 to 1.05] | 0.36 |
| PEARL | 1.02 [0.98 to 1.07] | 0.25 |
| PAZ | 0.98 [0.93 to 1.05] | 0.64 |
|  |  |  |

**Table S2:** Single nucleotide polymorphisms with p-value <0.05 in the *constant effect* pooled analysis from the Discovery Cohorts.

| SNP | Position | Regulome  DB | Westra et al.  cis p-value (gene) | Pooled ES [95% CI] | p-value | I^2^ |
| --- | --- | --- | --- | --- | --- | --- |
| rs66752327 | 40292462 | 6 | - | 0.81 [0.66 to 0.998] | 0.049 | 0 |
| rs6878901 | 40293177 | - | - | 0.81 [0.66 to 0.995] | 0.046 | 0 |
| rs348588 | 40295413 | - | - | 0.79 [0.66 to 0.98] | 0.037 | 0 |
| rs348571 | 40303985 | - | - | 0.81 [0.66 to 0.995] | 0.046 | 0.03 |
| rs12519882 | 40305221 | 6 | - | 0.81 [0.66 to 0.995] | 0.046 | 0 |
| rs4340954 | 40306777 | 6 | - | 0.81 [0.66 to 0.995] | 0.045 | 0 |
| rs348561 | 40312392 | 5 | - | 0.81 [0.66 to 0.995] | 0.046 | 0.03 |
| rs115430320 | 40318274 | - | - | 0.55 [0.39 to 0.79] | 0.001 | 0 |
| rs348601 | 40320006 | 5 | - | 0.87 [0.74 to 0.9995] | 0.049 | 0 |
| rs437342 | 40320992 | 6 | - | 0.76 [0.63 to 0.91] | 0.003 | 0 |
| rs348599 | 40321158 | 6 | - | 0.76 [0.63 to 0.91] | 0.003 | 0 |
| rs348597 | 40322184 | 5 | - | 0.76 [0.63 to 0.91] | 0.003 | 0 |
| rs348594 | 40323938 | 6 | - | 0.85 [0.74 to 0.995] | 0.045 | 0.0 |
| rs12173214 | 40325235 | 5 | - | 0.76 [0.63 to 0.91] | 0.003 | 0 |
| rs2034185 | 40325658 | - | - | 0.76 [0.63 to 0.91] | 0.003 | 0 |
| rs379109 | 40326387 | 6 | - | 0.76 [0.63 to 0.91] | 0.003 | 0 |
| rs433817 | 40327301 | 6 | - | 0.76 [0.63 to 0.93] | 0.006 | 0 |
| rs422083 | 40328070 | 5 | - | 0.78 [0.63 to 0.93] | 0.010 | 0 |
| rs443583 | 40333083 | 4 | - | 0.76 [0.63 to 0.91] | 0.003 | 0 |
| rs436695 | 40333672 | 4 | - | 0.76 [0.63 to 0.91] | 0.003 | 0 |
| rs394213 | 40333751 | 3a | - | 0.76 [0.63 to 0.91] | 0.003 | 0 |
| rs76460482 | 40344081 | - | - | 0.76 [0.6 to 0.95] | 0.017 | 0.24 |
| rs35870239 | 40344952 | - | - | 0.74 [0.59 to 0.93] | 0.009 | 0.15 |
| rs1550762 | 40346836 | 5 | - | 0.81 [0.66 to 0.98] | 0.029 | 0.36 |
| rs16869831 | 40347259 | 5 | - | 0.81 [0.68 to 0.99] | 0.043 | 0.37 |
| rs16869833 | 40347574 | - | - | 0.81 [0.66 to 0.98] | 0.029 | 0.36 |
| rs57869926 | 40349002 | 6 | - | 0.81 [0.66 to 0.98] | 0.029 | 0.36 |
| rs62359777 | 40350759 | 6 | - | 0.81 [0.68 to 0.95] | 0.012 | 0 |
| rs73082837 | 40354057 | 5 | - | 1.2 [1.02 to 1.41] | 0.033 | 0 |
| rs73082854 | 40357812 | 4 | - | 1.2 [1.02 to 1.41] | 0.036 | 0 |
| rs200255041 | 40358115 | - | - | 0.81 [0.68 to 0.99] | 0.037 | 0.31 |
| rs12517783 | 40360140 | 5 | - | 0.81 [0.68 to 0.99] | 0.043 | 0.26 |
| rs34385629 | 40360360 | 5 | - | 0.83 [0.68 to 0.995] | 0.045 | 0.26 |
| rs62359397 | 40360783 | - | - | 0.81 [0.68 to 0.99] | 0.038 | 0.29 |
| rs12513399 | 40361668 | 5 | - | 0.81 [0.68 to 0.99] | 0.043 | 0.26 |
| rs12518585 | 40361676 | 5 | - | 1 [0.68 to 0.99] | 0.042 | 0.27 |
| rs4957277 | 40361734 | 5 | - | 0.83 [0.68 to 0.998] | 0.048 | 0.24 |
| rs4957278 | 40361820 | 5 | - | 0.81 [0.68 to 0.995] | 0.044 | 0.26 |
| rs56157819 | 40362387 | - | - | 0.83 [0.68 to 0.998] | 0.047 | 0.29 |
| rs79724544 | 40363315 | 5 | - | 0.71 [0.56 to 0.9] | 0.005 | 0.3 |
| rs59830931 | 40363541 | - | - | 0.81 [0.68 to 0.99] | 0.043 | 0.26 |
| rs6891334 | 40365531 | 5 | - | 1.2 [1.01 to 1.41] | 0.041 | 0 |
| rs75248677 | 40365721 | 5 | - | 0.71 [0.55 to 0.89] | 0.005 | 0.38 |
| rs12514679 | 40366803 | 6 | - | 0.81 [0.68 to 0.99] | 0.038 | 0.29 |
| rs10941504 | 40367355 | 5 | - | 0.81 [0.68 to 0.98] | 0.033 | 0.33 |
| rs56952742 | 40368332 | - | - | 0.81 [0.68 to 0.99] | 0.043 | 0.26 |
| rs12055323 | 40368894 | 6 | - | 0.81 [0.68 to 0.99] | 0.043 | 0.26 |
| rs10462011 | 40372062 | - | - | 0.81 [0.68 to 0.99] | 0.042 | 0.27 |
| rs7725523 | 40372223 | - | - | 0.81 [0.69 to 0.95] | 0.017 | 0 |
| rs12517921 | 40375907 | 5 | - | 0.81 [0.68 to 0.99] | 0.043 | 0.26 |
| rs56253044 | 40376129 | 6 | - | 0.81 [0.68 to 0.98] | 0.032 | 0.33 |
| rs56049341 | 40376614 | 5 | - | 2.45 [1.29 to 4.68] | 0.007 | 0 |
| rs12516012 | 40377342 | 5 | - | 0.81 [0.68 to 0.98] | 0.034 | 0.30 |
| rs16869860 | 40377631 | - | - | 0.81 [0.68 to 0.98] | 0.032 | 0.33 |
| rs56249542 | 40378309 | 5 | - | 0.81 [0.68 to 0.98] | 0.033 | 0.33 |
| rs1031168 | 40381620 | 6 | - | 0.81 [0.69 to 0.95] | 0.017 | 0 |
| rs56027413 | 40382134 | 2b | - | 1.17 [1.002 to 1.41] | 0.046 | 0 |
| rs12520940 | 40382428 | 4 | - | 0.81 [0.68 to 0.98] | 0.033 | 0.33 |
| rs16869876 | 40383139 | 4 | - | 0.81 [0.68 to 0.99] | 0.038 | 0.33 |
| rs56353117 | 40383701 | 5 | - | 0.81 [0.68 to 0.98] | 0.033 | 0.33 |
| rs7707931 | 40496930 | 6 | - | 0.79 [0.66 to 0.98] | 0.026 | 0 |
| rs1876140 | 40505752 | - | 3.68x10^-15^ (*PTGER4*) | 0.79 [0.66 to 0.98] | 0.026 | 0 |
| rs13171949 | 40509008 | - | - | 0.79 [0.66 to 0.98] | 0.026 | 0 |
| rs6879508 | 40516303 | 5 | - | 0.79 [0.66 to 0.95] | 0.019 | 0 |
| rs6880662 | 40516860 | - | - | 0.79 [0.66 to 0.95] | 0.019 | 0 |
| rs7736920 | 40520217 | - | 7.20x10^-13^ (*PTGER4*) | 0.79 [0.65 to 0.95] | 0.016 | 0 |
| rs7737053 | 40520221 | - | - | 0.79 [0.65 to 0.95] | 0.017 | 0 |
| rs1876143 | 40521648 | 2b | 7.32x10^-13^ (*PTGER4*) | 0.79 [0.66 to 0.95] | 0.019 | 0 |
| rs7718129 | 40528679 | - | 6.94x10^-13^ (*PTGER4*) | 0.79 [0.66 to 0.95] | 0.021 | 0 |
| rs7718309 | 40528899 | - | 6.94x10^-13^ (*PTGER4*) | 0.79 [0.66 to 0.95] | 0.019 | 0 |
| rs954687 | 40529709 | 6 | 2.04x10^-12^ (*PTGER4*) | 0.78 [0.65 to 0.93] | 0.011 | 0 |
| rs6870587 | 40530699 | 6 | 2.05x10^-12^ (*PTGER4*) | 0.78 [0.65 to 0.93] | 0.011 | 0 |
| rs4957319 | 40532615 | - | 2.08x10^-12^ (*PTGER4*) | 0.78 [0.65 to 0.93] | 0.011 | 0 |
| rs969258 | 40549754 | 5 | 8.10x10^-13^ (*PTGER4*) | 0.79 [0.66 to 0.95] | 0.016 | 0 |
| rs7713972 | 40552474 | 4 | - | 0.79 [0.66 to 0.95] | 0.018 | 0 |
| rs6864749 | 40568593 | 5 | - | 0.79 [0.66 to 0.95] | 0.016 | 0 |
| rs10055946 | 40570075 | 4 | 3.74x10^-13^ (*PTGER4*) | 0.79 [0.66 to 0.95] | 0.016 | 0 |
|  |  |  |  |  |  |  |

CI: Confidence interval, ES: Effect size, SNP: Single nucleotide polymorphism.

**Table S3**: Single nucleotide polymorphisms with p-value <0.05 in the *time-varying effect* pooled analysis from the Discovery Cohorts.

| SNP | Position | Regulome  DB | Westra et al.  cis p-value (gene) | Pooled ES [95% CI] | p-value | I^2^ |
| --- | --- | --- | --- | --- | --- | --- |
| rs1550762 | 40346836 | 5 | - | 1.05 [1.01 to 1.07] | 0.02 | 0 |
| rs16869831 | 40347259 | 5 | - | 1.02 [1.005 to 1.07] | 0.02 | 0 |
| rs16869833 | 40347574 | - | - | 1.05 [1.01 to 1.07] | 0.02 | 0 |
| rs62359774 | 40348218 | 5 | - | 1.05 [1.01 to 1.07] | 0.01 | 0 |
| rs57869926 | 40349002 | 6 | - | 1.05 [1.01 to 1.07] | 0.02 | 0 |
| rs4286720 | 40349616 | - | - | 1.02 [1.005 to 1.07] | 0.02 | 0 |
| rs80058440 | 40350930 | 6 | - | 1.05 [1.01 to 1.07] | 0.01 | 0 |
| rs7725639 | 40355836 | - | - | 0.98 [0.95 to 0.99] | 0.01 | 0.15 |
| rs62359395 | 40356477 | 5 | - | 1.02 [1 to 1.07] | 0.02 | 0 |
| rs6451489 | 40357663 | 5 | - | 0.98 [0.95 to 0.99] | 0.01 | 0.15 |
| rs73082857 | 40357946 | 5 | - | 0.98 [0.95 to 0.99] | 0.02 | 0.08 |
| rs200255041 | 40358115 | - | - | 1.05 [1.01 to 1.09] | 0.02 | 0 |
| rs77269908 | 40358438 | - | - | 1.02 [1.002 to 1.07] | 0.04 | 0 |
| rs12517783 | 40360140 | 5 | - | 1.05 [1.01 to 1.07] | 0.01 | 0 |
| rs34385629 | 40360360 | 5 | - | 1.05 [1.01 to 1.07] | 0.02 | 0 |
| rs62359397 | 40360783 | - | - | 1.05 [1.01 to 1.07] | 0.01 | 0 |
| rs12523599 | 40361336 | 6 | - | 1.02 [1.005 to 1.07] | 0.02 | 0 |
| rs12513399 | 40361668 | 5 | - | 1.05 [1.01 to 1.07] | 0.01 | 0 |
| rs12518585 | 40361676 | 5 | - | 1.05 [1.01 to 1.07] | 0.01 | 0 |
| rs4957277 | 40361734 | 5 | - | 1.05 [1.01 to 1.07] | 0.01 | 0 |
| rs4957278 | 40361820 | 5 | - | 1.05 [1.01 to 1.07] | 0.01 | 0 |
| rs56157819 | 40362387 | - | - | 1.05 [1.01 to 1.07] | 0.01 | 0 |
| rs59830931 | 40363541 | - | - | 1.05 [1.01 to 1.07] | 0.01 | 0 |
| rs12514679 | 40366803 | 6 | - | 1.05 [1.01 to 1.07] | 0.01 | 0 |
| rs10941504 | 40367355 | 5 | - | 1.05 [1.01 to 1.07] | 0.01 | 0 |
| rs56952742 | 40368332 | - | - | 1.05 [1.01 to 1.07] | 0.01 | 0 |
| rs12055323 | 40368894 | 6 | - | 1.05 [1.01 to 1.07] | 0.01 | 0 |
| rs10462011 | 40372062 | - | - | 1.05 [1.01 to 1.07] | 0.01 | 0 |
| rs72745909 | 40375047 | 4 | - | 1.05 [1.01 to 1.07] | 0.01 | 0 |
| rs12517921 | 40375907 | 5 | - | 1.05 [1.01 to 1.07] | 0.01 | 0 |
| rs56253044 | 40376129 | 6 | - | 1.05 [1.01 to 1.07] | 0.01 | 0 |
| rs12515934 | 40376930 | 2b | - | 1.05 [1.01 to 1.07] | 0.004 | 0.27 |
| rs12516012 | 40377342 | 5 | - | 1.05 [1.01 to 1.07] | 0.01 | 0 |
| rs16869860 | 40377631 | - | - | 1.05 [1.01 to 1.07] | 0.01 | 0 |
| rs56249542 | 40378309 | 5 | - | 1.05 [1.01 to 1.07] | 0.01 | 0 |
| rs12520940 | 40382428 | 4 | - | 1.05 [1.01 to 1.07] | 0.01 | 0 |
| rs16869876 | 40383139 | 4 | - | 1.05 [1.01 to 1.07] | 0.01 | 0 |
| rs56353117 | 40383701 | 5 | - | 1.05 [1.01 to 1.07] | 0.01 | 0 |
| rs2120856 | 40388450 | 5 | - | 1.05 [1.01 to 1.07] | 0.01 | 0.35 |
| rs13160782 | 40428061 | 4 | - | 1.05 [1.01 to 1.07] | 0.01 | 0 |
| rs13186880 | 40429429 | - | - | 1.05 [1.01 to 1.07] | 0.01 | 0 |
| rs13165432 | 40441645 | 5 | - | 1.05 [1.01 to 1.07] | 0.01 | 0 |
| rs76523431 | 40443657 | 6 | - | 1.1 [1.01 to 1.2] | 0.02 | 0.36 |
| rs78607701 | 40447491 | 4 | - | 0.93 [0.89 to 0.98] | 0.004 | 0 |
| rs75502182 | 40476835 | 6 | - | 0.98 [0.93 to 0.995] | 0.02 | 0 |
| rs76798728 | 40480404 | 4 | - | 0.93 [0.89 to 0.98] | 0.001 | 0 |
| rs58752461 | 40481432 | 4 | - | 0.98 [0.93 to 0.998] | 0.03 | 0 |
| rs78053945 | 40484493 | - | - | 0.98 [0.93 to 0.995] | 0.02 | 0 |
| rs77676983 | 40491596 | 5 | - | 0.98 [0.93 to 0.995] | 0.03 | 0 |
| rs4587119 | 40492734 | - | 1.16x10^-16^ (*PTGER4*) | 1.05 [1.005 to 1.07] | 0.03 | 0.31 |
| rs13181935 | 40493646 | - | 6.88 x10^-15^ (*PTGER4*) | 1.05 [1.01 to 1.1] | 0.01 | 0 |
| rs7707931 | 40496930 | 6 | 3.68 x10^-13^ (*PTGER4*) | 1.02 [1.005 to 1.07] | 0.02 | 0 |
| rs74630635 | 40510535 | - | - | 1.05 [1.01 to 1.1] | 0.02 | 0 |
| rs7708224 | 40579767 | - | 7.18x10^-13^ (*PTGER4*) | 1.05 [1.01 to 1.07] | 0.03 | 0.49 |
| rs7705019 | 40587804 | 3a | - | 1.05 [1.01 to 1.07] | 0.03 | 0.49 |
| rs2036596 | 40588374 | 6 | - | 1.05 [1.01 to 1.07] | 0.05 | 0.66 |
| rs9647540 | 40588918 | - | - | 1.05 [1.01 to 1.07] | 0.03 | 0.49 |
| rs7711611 | 40592768 | - | 1.18x10^-11^ (*PTGER4*) | 1.05 [1.01 to 1.07] | 0.03 | 0.46 |
| rs9292783 | 40594758 | - | 1.76x10^-11^ (*PTGER4*) | 1.05 [1.01 to 1.07] | 0.03 | 0.47 |
| rs6878946 | 40595404 | - | 1.69x10^-11^ (*PTGER4*) | 1.05 [1.01 to 1.07] | 0.03 | 0.48 |
| rs9986289 | 40596247 | 6 | 3.39x10^-11^ (*PTGER4*) | 1.05 [1.01 to 1.07] | 0.03 | 0.47 |
| rs1505991 | 40597754 | - | 6.07x10^-11^ (*PTGER4*) | 1.05 [1.01 to 1.07] | 0.01 | 0.37 |
| rs7710376 | 40597896 | 5 | 9.98x10^-11^ (*PTGER4*) | 1.05 [1.01 to 1.07] | 0.01 | 0.37 |
| rs7710825 | 40598018 | 5 | 6.92x10^-11^ (*PTGER4*) | 1.05 [1.01 to 1.07] | 0.02 | 0.23 |
| rs4434422 | 40600917 | 5 | 5.93x10^-11^ (*PTGER4*) | 1.05 [1.01 to 1.07] | 0.01 | 0.37 |
| rs6892586 | 40601695 | 5 | 5.93x10^-11^ (*PTGER4*) | 1.05 [1.01 to 1.07] | 0.01 | 0.37 |
| rs6892791 | 40601799 | 5 | 5.93x10^-11^ (*PTGER4*) | 1.05 [1.01 to 1.07] | 0.01 | 0.37 |
| rs2174550 | 40602383 | - | - | 0.93 [0.87 to 0.995] | 0.04 | 0.33 |
| rs4409138 | 40603605 | 2b | - | 1.05 [1.01 to 1.07] | 0.01 | 0.37 |
| rs6890899 | 40607041 | - | 5.83x10^-11^ (*PTGER4*) | 1.05 [1.01 to 1.07] | 0.01 | 0.29 |
| rs13181692 | 40607998 | 5 | 8.14x10^-11^ (*PTGER4*) | 1.05 [1.01 to 1.07] | 0.01 | 0.30 |
| rs4957324 | 40611975 | 6 | 5.32x10^-11^ (*PTGER4*) | 1.05 [1.01 to 1.07] | 0.01 | 0.26 |
| rs924967 | 40615122 | - | 2.36x10^-11^ (*PTGER4*) | 1.05 [1.01 to 1.07] | 0.01 | 0.26 |
| rs4957325 | 40620582 | 5 | - | 1.05 [1.01 to 1.07] | 0.01 | 0.32 |
| rs4957137 | 40620793 | 5 | - | 1.05 [1.01 to 1.07] | 0.01 | 0.26 |
| rs1354674 | 40623128 | 2b | - | 1.05 [1.005 to 1.07] | 0.02 | 0 |
| rs76504641 | 40624582 | 6 | - | 0.95 [0.93 to 0.99] | 0.02 | 0 |
| rs11955175 | 40626753 | - | 0.001 (*PTGER4*) | 0.92 [0.89 to 0.98] | 0.01 | 0 |
| rs112239156 | 40649478 | 5 | - | 0.93 [0.89 to 0.98] | 0.01 | 0 |
| rs11960585 | 40662007 | - | - | 0.95 [0.91 to 0.99] | 0.01 | 0 |
| rs79132719 | 40668525 | 6 | - | 0.95 [0.91 to 0.99] | 0.01 | 0 |
| rs4432939 | 40671099 | - | - | 0.98 [0.95 to 0.998] | 0.03 | 0 |
| rs79575541 | 40672422 | 5 | - | 0.95 [0.91 to 0.99] | 0.01 | 0 |
| rs78426328 | 40673176 | - | - | 0.95 [0.91 to 0.99] | 0.01 | 0 |
| rs11953977 | 40674690 | 5 | - | 0.95 [0.91 to 0.99] | 0.01 | 0 |
| rs11953942 | 40674713 | 5 | - | 0.95 [0.91 to 0.99] | 0.01 | 0 |
| rs11954639 | 40674979 | 4 | 0.002 (*PTGER4*) | 0.95 [0.91 to 0.99] | 0.01 | 0 |
| rs11956741 | 40677576 | 4 | - | 0.95 [0.91 to 0.99] | 0.01 | 0 |
| rs45480797 | 40680964 | 2a | - | 0.95 [0.91 to 0.99] | 0.01 | 0 |
| rs2228058 | 40681254 | 4 | 0.002 (*PTGER4*) | 0.95 [0.91 to 0.99] | 0.02 | 0 |
| rs78733746 | 40727730 | 4 | - | 0.93 [0.87 to 0.99] | 0.02 | 0 |
|  |  |  |  |  |  |  |

CI: Confidence interval, ES: Effect size, SNP: Single nucleotide polymorphism.

**Table S4**: Single nucleotide polymorphisms selected for replication in the *constant effect* analysis, and their associated with radiological joint damage in the 6 Cohorts.

|  |  | HCSC-RAC | | PAZ | | PEARL | | EAC | | NDB | | Wichita | |
| --- | --- | --- | --- | --- | --- | --- | --- | --- | --- | --- | --- | --- | --- |
| SNP | Position | ES  [95% CI] | p | ES  [95% CI] | p | ES  [95% CI] | p | ES  [95% CI] | p | ES  [95% CI] | p | ES  [95% CI] | p |
| rs348561 | 40312392 | 0.81  [0.63 to 1.03] | 0.09 | 0.61  [0.34 to 1.09] | 0.09 | 1.02  [0.65 to 1.62] | 0.92 | 0.96  [0.83 to 1.10] | 0.52 | 1.03  [0.87 to 1.23] | 0.71 | - | - |
| rs115430320 | 40318274 | 0.56  [0.36 to 0.88] | 0.01 | 0.49  [0.06 to 3.78] | 0.48 | 0.54  [0.27 to 1.08] | 0.08 | 0.84  [0.65 to 1.08] | 0.18 | 1.03  [0.76 to 1.40] | 0.83 | 1.32  [0.8 to 2.18] | 0.26 |
| rs348601 | 40320006 | 0.85  [0.70 to 1.02] | 0.09 | 0.7  [0.46 to 1.08] | 0.10 | 0.99  [0.74 to 1.32] | 0.96 | 0.89  [0.80 to 0.99] | 0.04 | 0.97  [0.85 to 1.10] | 0.63 | 1.13  [0.89 to 1.44] | 0.31 |
| rs394213 | 40333751 | 0.75  [0.59 to 0.95] | 0.02 | 0.62  [0.37 to 1.06] | 0.08 | 0.86  [0.59 to 1.25] | 0.43 | 0.92  [0.81 to 1.04] | 0.18 | 1.01  [0.87 to 1.18] | 0.88 | 1.05  [0.81 to 1.34] | 0.72 |
| rs35870239 | 40344952 | 0.69  [0.52 to 0.91] | 0.01 | 0.65  [0.36 to 1.15] | 0.13 | 1.07  [0.63 to 1.82] | 0.80 | 0.94  [0.81 to 1.09] | 0.42 | 1.01  [0.84 to 1.22] | 0.89 | 1.06  [0.77 to 1.45] | 0.73 |
| rs62359777 | 40350759 | 0.76  [0.62 to 0.94] | 0.01 | 0.71  [0.44 to 1.16] | 0.17 | 1.003  [0.70 to 1.44] | 0.99 | 0.96  [0.86 to 1.08] | 0.50 | - | - | - | - |
| rs75248677 | 40365721 | 0.67  [0.48 to 0.94] | 0.02 | 0.63  [0.42 to 0.94] | 0.02 | 1.29  [0.63 to 2.64] | 0.48 | 0.97  [0.82 to 1.14] | 0.68 | 0.93  [0.75 to 1.17] | 0.56 | 0.89  [0.65 to 1.21] | 0.45 |
| rs56049341 | 40376614 | 2.86  [1.14 to 7.16] | 0.03 | 2.22  [0.4 to 12.38] | 0.36 | 2.02  [0.67 to 6.14] | 0.21 | 1.34  [0.72 to 2.51] | 0.36 | - | - | - | - |
| rs56027413 | 40382134 | 1.17  [0.93 to 1.47] | 0.19 | 1.32  [0.89 to 1.97] | 0.16 | 1.13  [0.82 to 1.56] | 0.44 | 1.03  [0.91 to 1.16] | 0.62 | 0.96  [0.83 to 1.12] | 0.63 | 1.22  [0.94 to 1.59] | 0.13 |
| rs12520940 | 40382428 | 0.73  [0.58 to 0.93] | 0.01 | 0.82  [0.5 to 1.35] | 0.43 | 1.13  [0.73 to 1.75] | 0.58 | 0.99  [0.87 to 1.13] | 0.87 | 0.99  [0.84 to 1.16] | 0.88 | 1.10  [0.83 to 1.46] | 0.49 |
| rs1876140 | 40505752 | 0.81  [0.63 to 1.04] | 0.10 | 0.97  [0.57 to 1.66] | 0.92 | 0.72  [0.49 to 1.04] | 0.08 | 1.07  [0.94 to 1.22] | 0.31 | 0.97  [0.83 to 1.14] | 0.75 | 1.41  [0.98 to 2.03] | 0.07 |
| rs1876143 | 40521648 | 0.82  [0.64 to 1.05] | 0.12 | 0.97  [0.57 to 1.66] | 0.92 | 0.68  [0.46 to 0.98] | 0.04 | 1.08  [0.95 to 1.23] | 0.26 | 0.99  [0.84 to 1.16] | 0.86 | 1.31  [0.92 to 1.88] | 0.14 |
|  |  |  |  |  |  |  |  |  |  |  |  |  |  |

CI: Confidence interval, ES: Effect size, SNP: Single nucleotide polymorphism.

**Table S5**: Single nucleotide polymorphisms selected for replication in the *time-varying effect* analysis, and their associated with radiological joint damage in the 6 Cohorts.

|  |  | HCSC-RAC | | PAZ | | PEARL | | EAC | | NDB | | Wichita | |
| --- | --- | --- | --- | --- | --- | --- | --- | --- | --- | --- | --- | --- | --- |
| SNP | Position | ES [95% CI] | p | ES [95% CI] | p | ES [95% CI] | p | ES [95% CI] | p | ES [95% CI] | p | ES [95% CI] | p |
| rs80058440 | 40350930 | 1.04  [0.99 to 1.09] | 0.07 | 1.06  [0.98 to 1.15] | 0.15 | 1.04  [0.96 to 1.14] | 0.35 | 0.99  [0.97 to 1.02] | 0.62 | - | - | - | - |
| rs6451489 | 40357663 | 0.97  [0.94 to 0.996] | 0.03 | 1.04  [0.94 to 1.14] | 0.45 | 0.96  [0.91 to 1.01] | 0.09 | 1.01  [0.99 to 1.02] | 0.46 | 0.99  [0.90 to 1.11] | 0.93 | 1.01  [0.97 to 1.04] | 0.66 |
| rs12515934 | 40376930 | 1.03  [0.99 to 1.07] | 0.15 | 1.10  [0.99 to 1.22] | 0.06 | 1.05  [0.96 to 1.15] | 0.32 | 1.01  [0.98 to 1.03] | 0.66 | 0.99  [0.86 to 1.15] | 0.93 | 1.01  [0.96 to 1.06] | 0.6 |
| rs12520940 | 40382428 | 1.03  [1.0003 to 1.07] | 0.05 | 1.05  [0.97 to 1.13] | 0.23 | 1.05  [0.98 to 1.12] | 0.2 | 0.99  [0.98 to 1.02] | 0.65 | 0.96  [0.86 to 1.08] | 0.48 | 0.98  [0.94 to 1.01] | 0.18 |
| rs13160782 | 40428061 | 1.04  [1.01 to 1.07] | 0.02 | 1.05  [0.96 to 1.14] | 0.24 | 1.02  [0.96 to 1.08] | 0.48 | 1.02  [1.001 to 1.04] | 0.04 | 0.99  [0.89 to 1.11] | 0.92 | 0.99  [0.95 to 1.02] | 0.47 |
| rs76523431 | 40443657 | 1.17  [1.05 to 1.30] | 0.01 | 0.93  [0.59 to 1.48] | 0.77 | 1.02  [0.90 to 1.16] | 0.74 | 1.08  [1.02 to 1.14] | 0.01 | 1.08  [0.79 to 1.47] | 0.64 | 1.21  [1.06 to 1.38] | 0.005 |
| rs78607701 | 40447491 | 0.93  [0.88 to 0.99] | 0.03 | 0.99  [0.79 to 1.24] | 0.91 | 0.93  [0.85 to 1.01] | 0.07 | 0.99  [0.95 to 1.04] | 0.75 | 0.94  [0.73 to 1.21] | 0.63 | 0.98  [0.90 to 1.05] | 0.53 |
| rs58752461 | 40481432 | 0.97  [0.94 to 1.004] | 0.08 | 0.98  [0.88 to 1.08] | 0.68 | 0.96  [0.91 to 1.02] | 0.22 | 0.98  [0.96 to 1.01] | 0.14 | 1.02  [0.88 to 1.19] | 0.76 | 0.97  [0.93 to 1.02] | 0.25 |
| rs4587119 | 40492734 | 1.03  [0.99 to 1.07] | 0.08 | 1.09  [1.004 to 1.19] | 0.04 | 0.98  [0.89 to 1.08] | 0.70 | 1.01  [0.98 to 1.04] | 0.49 | 0.98  [0.84 to 1.14] | 0.77 | 0.98  [0.93 to 1.03] | 0.42 |
| rs13181935 | 40493646 | 1.04  [1.0004 to 1.09] | 0.05 | 1.09  [1.004 to 1.19] | 0.04 | 1.002  [0.90 to 1.12] | 0.97 | 1.01  [0.98 to 1.04] | 0.35 | 0.97  [0.83 to 1.15] | 0.76 | 0.98  [0.93 to 1.04] | 0.56 |
| rs7707931 | 40496930 | 1.04  [1.005 to 1.07] | 0.03 | 1.04  [0.97 to 1.13] | 0.26 | 1.0004  [0.94 to 1.06] | 0.99 | 1.01  [0.99 to 1.03] | 0.34 | 0.96  [0.85 to 1.08] | 0.5 | 0.99  [0.94 to 1.04] | 0.61 |
| rs74630635 | 40510535 | 1.04  [0.98 to 1.10] | 0.18 | 1.10  [1.003 to 1.21] | 0.04 | 1.06  [0.89 to 1.26] | 0.49 | 1.06  [1.02 to 1.11] | 0.005 | 0.89  [0.70 to 1.14] | 0.36 | 1.01  [0.90 to 1.12] | 0.89 |
| rs7705019 | 40587804 | 1.02  [0.98 to 1.06] | 0.25 | 1.09  [1.004 to 1.19] | 0.04 | 1.10  [1.01 to 1.20] | 0.03 | 1.02  [0.99 to 1.04] | 0.23 | 0.97  [0.84 to 1.12] | 0.67 | 0.99  [0.93 to 1.04] | 0.64 |
| rs2174550 | 40602383 | 0.94  [0.86 to 1.03] | 0.16 | 0.86  [0.74 to 0.99] | 0.04 | 1.004  [0.89 to 1.14] | 0.95 | 1.02  [0.99 to 1.05] | 0.31 | 0.91  [0.73 to 1.14] | 0.41 | 0.96  [0.90 to 1.02] | 0.17 |
| rs4409138 | 40603605 | 1.02  [0.99 to 1.06] | 0.23 | 1.09  [1.004 to 1.19] | 0.04 | 1.08  [0.996 to 1.18] | 0.06 | 1.01  [0.99 to 1.04] | 0.24 | 0.96  [0.83 to 1.10] | 0.52 | 0.99  [0.94 to 1.05] | 0.90 |
| rs924967 | 40615122 | 1.02  [0.99 to 1.06] | 0.23 | 1.09  [1.004 to 1.19] | 0.04 | 1.07  [0.99 to 1.15] | 0.10 | 1.01  [0.99 to 1.04] | 0.24 | 0.95  [0.83 to 1.09] | 0.45 | 0.99  [0.94 to 1.05] | 0.90 |
| rs76504641 | 40624582 | 0.97  [0.93 to 1.01] | 0.15 | 0.90  [0.80 to 0.99] | 0.05 | 0.97  [0.89 to 1.05] | 0.44 | 1.01  [0.98 to 1.03] | 0.61 | 0.93  [0.81 to 1.07] | 0.30 | 0.98  [0.94 to 1.03] | 0.48 |
| rs4432939 | 40671099 | 0.97  [0.94 to 1.004] | 0.09 | 0.96  [0.89 to 1.03] | 0.22 | 0.98  [0.93 to 1.03] | 0.44 | 0.98  [0.96 to 1.001] | 0.06 | 1.02  [0.89 to 1.17] | 0.73 | 0.98  [0.94 to 1.03] | 0.38 |
| rs45480797 | 40680964 | 0.95  [0.91 to 1.01] | 0.09 | 0.93  [0.80 to 1.09] | 0.35 | 0.93  [0.84 to 1.02] | 0.10 | 0.99  [0.96 to 1.02] | 0.38 | 0.97  [0.81 to 1.17] | 0.76 | 0.96  [0.89 to 1.05] | 0.36 |
| rs78733746 | 40727730 | 0.95  [0.88 to 1.02] | 0.17 | 0.90  [0.77 to 1.06] | 0.19 | 0.92  [0.82 to 1.02] | 0.11 | 1.004  [0.96 to 1.05] | 0.85 | 0.96  [0.74 to 1.26] | 0.79 | 0.99  [0.88 to 1.11] | 0.87 |
|  |  |  |  |  |  |  |  |  |  |  |  |  |  |

CI: Confidence interval, ES: Effect size, SNP: Single nucleotide polymorphism.
